# Anterolateral ligament of the knee: a step-by-step dissection

**DOI:** 10.1186/s12891-019-2517-0

**Published:** 2019-04-04

**Authors:** Diego Ariel de Lima, Camilo Partezani Helito, Matthew Daggett, Francisco Magalhães Monteiro Neto, Lana Lacerda de Lima, José Alberto Dias Leite, Maria Luzete Costa Cavalcante

**Affiliations:** 1 UFERSA. Universidade Federal Rural do Semi-Árido, Av João da Escóssia, 1300, Mossoró, RN CEP: 59607-330 Brazil; 2 USP. Grupo de Joelho, Instituto de Ortopedia e Traumatologia, Hospital das Clínicas HCFMUSP, Faculdade de Medicina da Universidade de São Paulo, São Paulo, Brazil; 3 Hospital Sírio Libanês, São Paulo, Brazil; 4 Kansas City University of Medicine and Biosciences, Kansas City, MO USA; 5 UFC. Departamento de Cirurgia da Universidade Federal do Ceará, Fortaleza, Brazil; 6 UERN. Universidade do Estado do Rio Grande do Norte, Mossoró, Brazil

## Abstract

**Background:**

The number of studies and clinical interest in the anterolateral ligament of the knee (ALL) has grown in recent years. A meticulous and accurate ALL dissection is vital in anatomic and biomechanical studies, and a standardized technique is not yet established. As such, the aim of this study was to describe a step-by-step ALL dissection technique that could help authors consistently identify the ALL.

**Methods:**

Twenty knees from frozen adult cadavers, with no preference for sex or age, were included in the study. All the cadavers were dissected using the same technique to determine the incidence of the ALL.

**Results:**

A transverse incision is performed in the iliotibial band (ITB), around 10 cm proximal to the topography of the lateral epicondyle of the femur. Next, the ITB undergoes anterograde blunt dissection until its insertion at Gerdy’s tubercle in the tibia. Maintaining biceps femoris insertion, a dissection is performed anteriorly to it, until the lateral collateral ligament (LCL) is found. Using the LCL, internal rotation and 30 to 60° flexion as references, the ALL can be located in the anterolateral topography of the knee, with its origin near the lateral epicondyle (proximal and posterior) and insertion between Gerdy’s tubercle and the fibula (4.0 mm to 7.0 mm below the tibial plateau), expanding to the lateral meniscus (between the body and anterior horn), exhibiting a mean length of 4.0 ± 0.4 cm and mean width of 5.5 ± 0.8 mm.

**Conclusions:**

The present article describes an effective and reproducible ALL dissection technique that made it was possible to identify the ligament in 100% of the cases in the present study.

**Electronic supplementary material:**

The online version of this article (10.1186/s12891-019-2517-0) contains supplementary material, which is available to authorized users.

## Background

Since the studies conducted by Vicent et al [1], Claes et al [2]. and Helito et al [3], the number of investigations and clinical interest in the anterolateral ligament of the knee (ALL) has risen in recent years [4, 5].

**Fig. 1:**
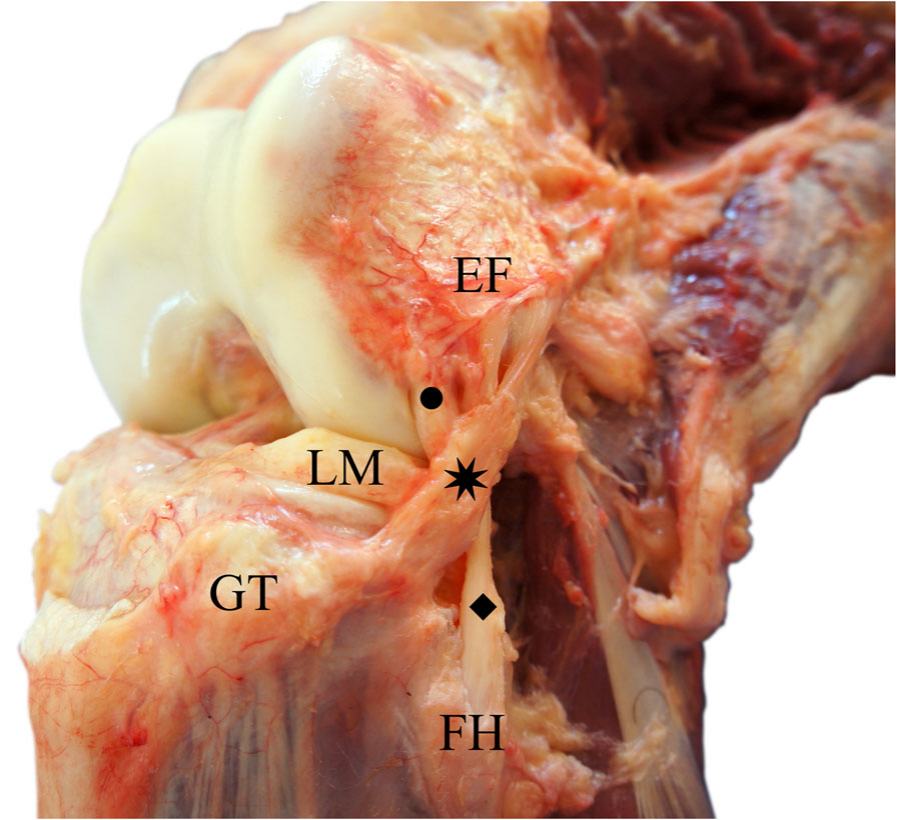
Anterolateral aspect of a right knee, showing the relationship between the anterolateral ligament, lateral collateral ligament and popliteus tendon. Eight pointed star (✷): Anterolateral Ligament; Diamond (◆): Lateral Collateral Ligament; Circle (●): Popliteus Tendon; FH: Fibular Head; GT: Gerdy’s Tubercle; LM: Lateral Meniscus; LE: Lateral Epicondyle of the Femur

The ALL is a triangular structure in the anterolateral topography of the knee found in a plane of dissection deep in relation to the iliotibial band (Fig. 1). It measures between 34 and 59 mm long, 4.0 mm and 7.0 mm high and around 2 mm thick in men and 1 mm in women. It originates near the lateral epicondyle of the femur and is inserted between Gerdy’s tubercle and the fibular head, expanding to the lateral meniscus [1–3, 6–9].

Despite ongoing controversy in the literature, the ALL apparently plays a role in anterolateral rotational stability of the knee, acting synergically to the anterior cruciate ligament, affecting the pivot shift in failed ACL surgery [6, 10–13]. Given this probable role in stability, the meticulous study of the anatomy and biomechanics of this ligament is vital. For this to occur, it is important that the ALL be dissected efficiently.

As such, the aim of the present study was to describe a step-by-step ALL dissection technique that may contribute to other research on the ligament in question.

## Methods

The study was approved by the Research Ethics Committee (CAAE: 78798617.5.0000.5049 - Brazil) and involved 20 knees from frozen adult cadavers, with no preference for sex or age. The cadavers were obtained from the Center for Forensic Studies of Ceará, Brazil (PEFOCE). The samples came from unclaimed cadavers after 30 days without contact from any relative or known, according to Local Law N° 8.501 of November 30, 1992.

Exclusion criteria were cadavers with signs of traumatic and/or degenerative injury that could hinder ALL dissection. However, none of the cadavers were excluded.

All the cadavers were dissected using the same technique in order to determine ALL incidence.

## Results (Additional file 1: Video S1)

The initial dissection consisted of ample exposure of the iliotibial band (ITB) after the skin and subcutaneous cell tissue were removed from the anterolateral surface of the knee. A transverse incision is made in the ITB, around 10 cm proximal to the topography of the lateral epicondyle of the femur. Next, the ITB undergoes antegrade blunt dissection until its insertion at Gerdy’s tubercle in the tibia. This step is vital and requires special care since the ALL adheres closely to the ITB. Although they are completely different and anatomically separated structures, the posterior deep part of the ITB might be confused with the ALL by some authors. This posterior deep part is known by many authors as “capsule-osseous” layer of the ITB and was subject of controversy during initial ALL studies. The differentiation between the ALL and the deep part of the ITB is not difficult, as the ITB has no clear attachment near the lateral epicondyle. The Kaplan fibers (attachment of the ITB to the distal femur) are proximal and not connected to the ALL femoral attachment [14–16]. Careless dissection may inadvertently injure the ALL, especially near the topography of the epicondyle.


**Additional file 1: Video S1** Dissection Technique. In the present video it is possible to observe the step-by-step dissection of the anterolateral ligament of the knee. (MP4 168452 kb)


**Fig. 2:**
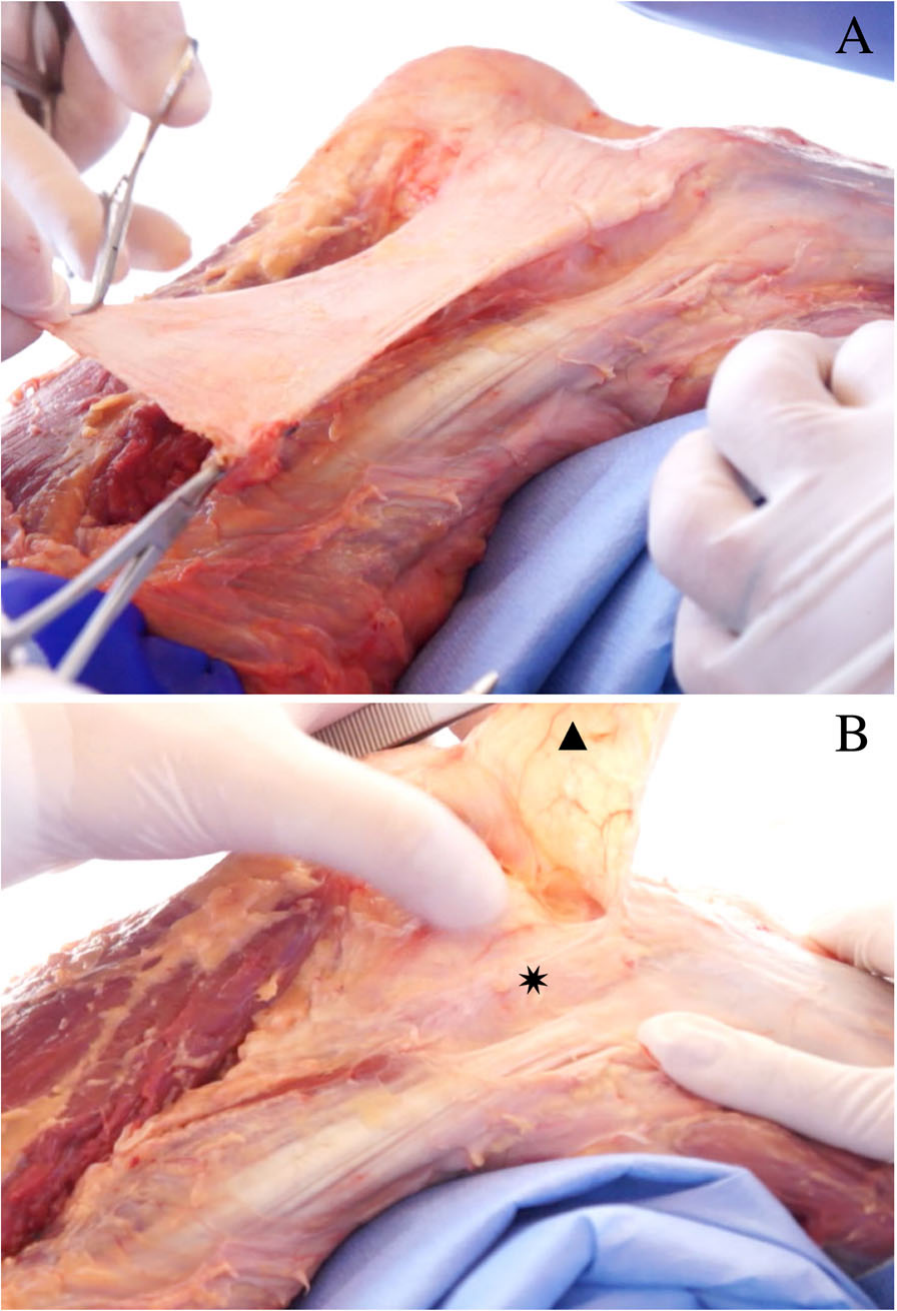
Antegrade dissection of the ITB. **a** ITB dissection until its insertion at Gerdy’s tubercle in the tibia. **b** ITB reflection and ALL fibers. Eight pointed star (✷): Anterolateral Ligament; Triangle (▲): Iliotibial Band

After reflection of the ITB, a structure composed of parallel fibers exhibiting an anteroinferior trajectory towards the anterolateral region of the tibia is visible. This structure is the ALL itself. With knee flexion between 30 and 60°, internal rotation stretches the ALL, making it easier to visualize (Fig. 2).

**Fig. 3:**
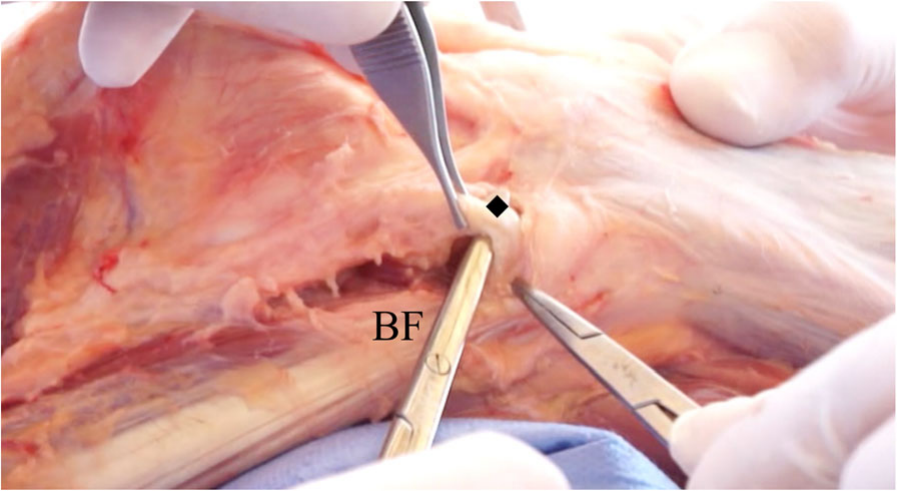
Dissection anterior to the biceps femoris and identification of the lateral collateral ligament. Diamond (◆): Lateral Collateral Ligament; BF: Biceps femoris

Next, a dissection is made between the anterior face of the biceps femoris muscle and posterior face of the recently visualized ALL. Some authors also call this recess as biceps-capsulo osseous iliotibial tract confluens and controversy regarding terminology also exists. Dieresis of this recess is performed until the lateral collateral ligament (LCL) is identified (Fig. 3).

**Fig. 4:**
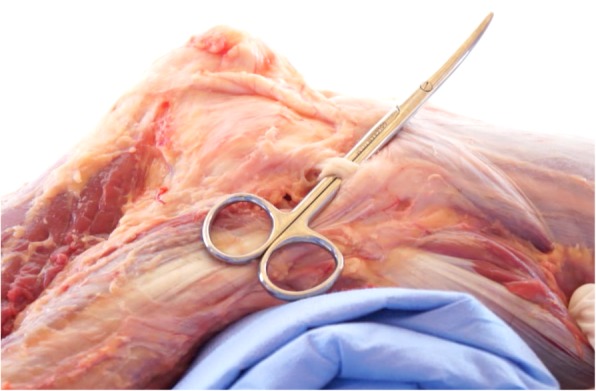
Lateral collateral ligament isolation

After the LCL is isolated (Fig. 4), dissection continues proximally to it until the origin of the ALL is identified, proximal to the lateral epicondyle of the femur and the origin of the LCL, maintaining a close relationship with the tendon of the popliteus muscle. This is another step that requires care, since the proximal segment of the ALL overlaps the LCL, and careless dissection could rupture the ALL fibers. Dissection continues distally immediately after the proximal portion of the ALL is isolated.

**Fig. 5:**
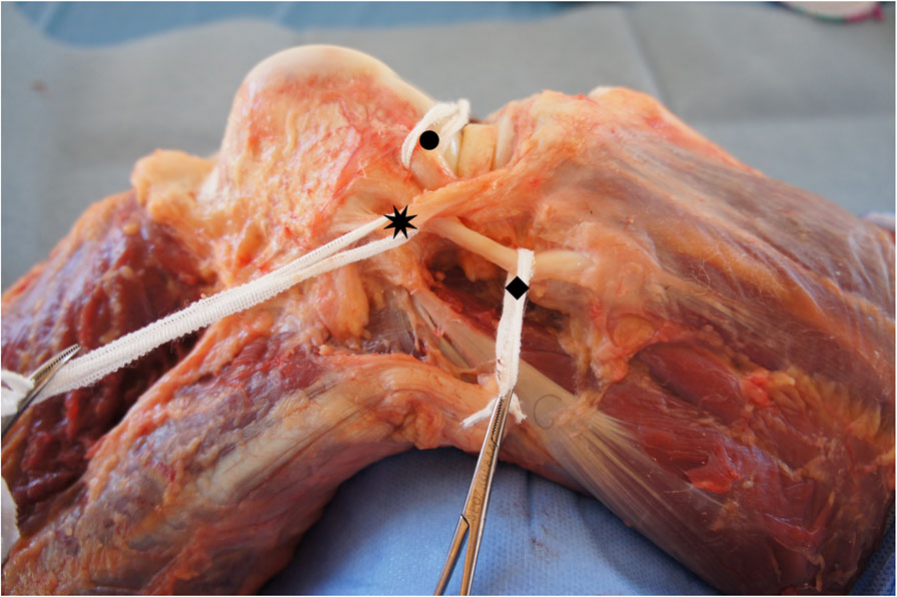
Isolation of the anterolateral ligament of the knee Eight pointed star (✷): Anterolateral Ligament; Diamond (◆): Lateral Collateral Ligament; Circle (●): Popliteus Tendon

As the dissection progresses, it is possible to identify the meniscal insertion, located between the body and the anterior horn of the lateral meniscus, as well as tibial insertion between the fibular head and Gerdy’s tubercle, 4.0 mm to 7.0 mm below the tibial plateau (Fig. 5).

The ALL was found in all 20 knees studied using the aforementioned technique, exhibiting a mean length of 4.0 ± 0.4 cm and mean width of 5.5 ± 0.8 mm. In all the cases, the ALL showed a proximal and posterior origin in relation to the lateral epicondyle of the femur. Also, in every case, it exhibited two insertions, one in the tibia and another on the periphery of the lateral meniscus, between the body and anterior horn.

## Discussion

Clinical and anatomic studies on the ALL have increased significantly in recent years. However, well performed, or even standardized dissections, are essential to achieving quality studies.

In 2016, Daggett et al. [17] described an ALL dissection technique consisting of 3 steps. Similarly, we performed an antegrade dissection of the ITB. However, in contrast to the Daggett et al. technique [17], we did not reflect the biceps femoris muscle posteriorly. We maintained biceps femoris insertion, dissecting anteriorly to it until we found the LCL.

Claes et al. [2] dissected 41 embalmed cadavers using antegrade dissection of the ITB and LCL, internal rotation and 60° flexion as references to find the ALL in the 3rd layer of the lateral aspect of the knee [18, 19]

Roessler et al. [20] described arthronomy using a lateral parapatellar approach. After tibial insertion of the ITB was identified, it was removed by retrograde dissection and reflection, leaving the fibrous connection of the ITB in the lateral femoral condyle intact. Roessler et al. [20] applied this technique to dissect 20 fresh cadavers, observing the ALL in 60% of the cases.

After dissecting skin and subcutaneous tissue, Helito et al. [3] performed tenotomy of the quadriceps tendon, arthrotomy using a medial parapatellar approach and osteotomy of the tibial tubercle to access the anterolateral region of the knee. Next, the iliotibial band was resected in Gerdy’s tubercle, followed by proximal and distal ALL isolation based on its original location close to the LCL. This technique obtained a 100% ALL visualization rate [3, 21, 22].

Fardin et al. [23] described a dissection technique starting with a section of the ITB 5 cm proximal to the lateral epicondyle of the femur, followed by detachment up to Gerdy’s tubercle in order to identify the articular capsule, the LCL and the tendon of the popliteus muscle. However, in contrast to literature data and evidence of the presence of the ALL is most knees, in none of the 15 specimens studied was the ALL identified properly. It is important to mention that Fardin et al. used embalmed specimens in their study.

Parker and Smith [24] dissected 53 embalmed cadavers and found the ALL in 96.2% of the cases. These authors described a similar technique to that reported here. They suggested using a technique with antegrade dissection of the ITB and LCL as a reference to locate the ALL. However, the main difference is that they only recommend searching for the ALL after total isolation of the LCL. Similarly, using the LCL as reference, Potu et al. [9] dissected cadavers fixed in formalin and found the ALL in only 4.6% of the cases. Due to the anterolateral path overlapping the proximal LCL fibers, if the LCL is completed isolated, the most proximal fibers of the ALL will need to be removed, what could affect is proper isolation and interfere in the dissection results.

With the passing of time and increasing number of published articles, the existence of the ALL is no longer the focus of studies. Understanding its function in knee kinematics is currently being investigated [4, 5]. However, a few studies still report a low ALL visualization rate in dissections [9, 23, 25–27]. Those that did not find the ALL were usually performed with techniques that might interfere with proper ALL isolation and on embalmed cadavers [9, 23, 26], which may hinder dissection, regardless of the technique used.

We do not know the extent to which the choice of technique influences ALL findings. However, as underscored by Daggett et al. [17], standardizing dissection techniques would be beneficial in terms of research and clinical-surgical practice.

## Conclusion

The present article describes an effective and reproducible ALL dissection technique that made it possible to identify the ALL in 100% of the cases in the present study.

## Abbreviations

ALL: Anterolateral ligament of the knee
CAAE: Research Ethics Committee Number
ITB: Iliotibial band
LCL: Lateral collateral ligament
PEFOCE: Forensic Studies of Ceará, Brazil
